# Case Report of Novel Genetic Variant in KCNT1 Channel and Pharmacological Treatment With Quinidine. Precision Medicine in Refractory Epilepsy

**DOI:** 10.3389/fphar.2021.648519

**Published:** 2021-05-28

**Authors:** M. C. Kravetz, M. S. Viola, J. Prenz, M. Curi, G. F. Bramuglia, S. Tenembaum

**Affiliations:** ^1^ Department of Pharmacology, Faculty of Farmacy and Biochemistry, University of Buenos Aires, Buenos Aires City, Argentina; ^2^ Department of Cardiology, Garrahan Hospital, Buenos Aires City, Argentina; ^3^ Fundacion Investigar, Buenos Aires City, Argentina; ^4^ Department of Neurology, Garrahan Hospital, Buenos Aires City, Argentina

**Keywords:** epilepsy, pharmacogenomics, pharmacokinetics, precision medicine, TDM, quinidine, HPLC

## Abstract

**Case introduction:** In this work we present a female infant patient with epilepsy of infancy with migrating focal seizures (EIMFS). Although many pharmacological schemes were attempted, she developed an encephalopathy with poor response to antiepileptic drugs and progressive cerebral dysfunction.

**Aim:** To present the pharmacological response and therapeutic drug monitoring of a paediatric patient with a severe encephalopathy carrying a genetic variant in KCNT1 gene, whose identification led to include quinidine (QND) in the treatment regimen as an antiepileptic drug.

**Case report:** Patient showed slow rhythmic activity (theta range) over left occipital areas with temporal propagation and oculo-clonic focal seizures and without tonic spasms three months after birth. At the age of 18 months showed severe impairments of motor and intellectual function with poor eye contact. When the patient was 4 years old, a genetic variant in the exon 24 of the KCNT1 gene was found. This led to the diagnosis of EIMFS. Due to antiepileptic treatment failed to control seizures, QND a KCNT1 blocker, was introduced as a therapeutic alternative besides topiramate (200 mg/day) and nitrazepam (2 mg/day). Therapeutic drug monitoring (TDM) of QND plasma levels needed to be implemented to establish individual therapeutic range and avoid toxicity. TDM for dose adjustment was performed to establish the individual therapeutic range of the patient. Seizures were under control with QND levels above 1.5 mcg/ml (65–70 mg/kg q. i.d). In addition, QND levels higher than 4.0 mcg/ml, were related to higher risk of suffering arrhythmia due to prolongation of QT segment. Despite initial intention to withdrawal topiramate completely, QND was no longer effective by itself and failed to maintain seizures control. Due to this necessary interaction between quinidine and topiramate, topiramate was stablished in a maintenance dose of 40 mg/day.

**Conclusion:** The implementation of Precision Medicine by using tools such as Next Generation Sequencing and TDM led to diagnose and select a targeted therapy for the treatment of a KCNT1-related epilepsy in a patient presented with EIMFS in early infancy and poor response to antiepileptic drugs. QND an old antiarrhythmic drug, due to its activity as KCNT1 channel blocker, associated to topiramate resulted in seizures control. Due to high variability observed in QND levels, TDM and pharmacokinetic characterization allowed to optimize drug regimen to maintain QND concentration between the individual therapeutic range and diminish toxicity.

**GRAPHICAL ABSTRACT:**
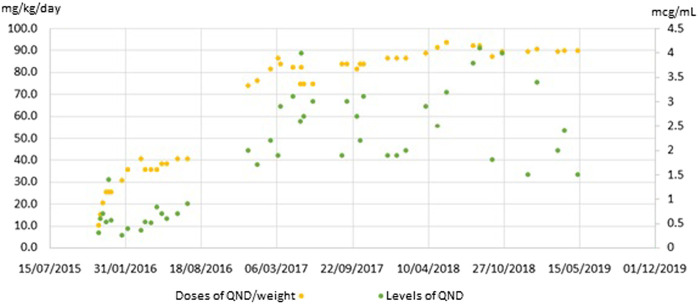


## Introduction

Epilepsy of infancy with migrating focal seizures (EIMFS) is a severe encephalopathy affecting children at very early stages. Seizures can begin as early as six months of life, and even a few weeks after delivery. This rare epileptic syndrome is characterized for pharmacological treatment resistance and poor prognosis. Its low prevalence varies from 1/200,000 to 1/1,000,000 habitants depending on of the population studied (Epilepsy of infancy with migrating focal seizures (2020) Epilepsy of infancy with migrating focal seizures | Epilepsy Action).

About etiology, the origin of this complex syndromes of infancy with migrating partial onset is not certain, but it is described the association with pathologic variants of KCNT1, SCN1A, SCN2A, PLCB1, TBC1D24 and CHD2 (International League Against Epilepsy, 2020 Epilepsy of infancy with migrating focal seizures, 2020). Around 70% of patients carries mutations in these and others genes and KCNT1 variants are implied in almost 40% of EIMFS (Lim et al., 2016).

*In vitro* functional studies have shown that KCNT1 carrying pathogenic variants generate larger currents when compared to wild-type KCNT1 channels, leading to the concept that a gain-of-function mechanism is responsible for epileptogenesis associated with KCNT1 variants (Barcia et al., 2012). Based on the ability to inhibit KCNT1 mutant channels, KCNT1 blockers like quinidine (QND), and old antiarrhythmic drug, and bepridil, have been proposed for the treatment of EIMFS (Milligan et al., 2014). Targeted therapy with QND has been introduced for compassionate use in EIMFS patients, resulting in variable anticonvulsant effect ranging from high pharmacological responses (Fukuoka et al., 2017) to a lack of efficacy or excessive toxicity (Chong et al., 2016; Madaan et al., 2018).

Previous data showed QND produced a concentration-dependent decrease in KCNT1 outward currents (Rizzo et al., 2016; Dilena et al., 2018) and that QND was even more potent in blocking KCNT1 channel carrying previously identified genetic variants, such as R950Q or E893K, in comparison with KCNT1 wild type (Dilena et al., 2018).

In this work we present a female pediatric patient with a severe case of EIMFS. Although many pharmacological schemes were attempted, the patient developed an epileptic syndrome with poor response to antiepileptic drugs and progressive cerebral dysfunction.

The patient had two episodes of apnea during the first week of life and showed poor eye contact, a trend toward a conjugate deviation of the eyes and head to the right or left, with horizontal nystagmus. She was extremely irritable. Three month later the patient was admitted due to three epileptic seizures recorded by electroencephalogram, which indicated slow rhythmic activity (theta range) over left occipital areas with temporal propagation. She presented oculo-clonic focal seizures which she repeated several times during examinations without tonic spasms.

The first year of life the patient received a drug regimen that included phenobarbital, valproic acid 750 mg/day, levetiracetam 600 mg/day, and ketogenic diet. Immunoglobulin therapy was also included because of low serum levels of Ig A and IgG.

As time progressed, the patient demonstrated spastic asymmetric quadriplegia, more prominent on the right, truncal hypotonia with poor head control, and lower-limb pyramidal tract signs. There were no laboratory abnormalities in blood nor CSF, and no sign of skeletal malformation on physical examination.

Electroencephalography during sleep (multiple recordings) demonstrated paroxysmal multifocal discharges with periods of electric attenuation or suppression which were not continuous. Electrographic seizures were also recorded, arising from the left frontal-temporal and right temporal-occipital regions. The clinical presentation and electroencephalographic features resembled an early myoclonic encephalopathy.

When the patient was 18 months old topiramate (TPM) was initiated that was related with a decrease in the frequency of seizures. At two years old (September 2011) seizures could be controlled receiving TPM 7 mg/kg/day, levetiracetam 48 mg/kg/day, and nitrazepam 2 mg/day (ketogenic diet was discontinuated). Nevertheless, electroencephalography during sleep in October 2011 revealed a typical sleep pattern with sleep spindles and vertex waves, in addition to infrequent sharp waves in left occipital region without generalized paroxysms or electric suppression.

A genomic study was performed, and no pathogenic variants were found in Glut1 gene, MCP2, CDKL5, FOXG1. Levetiracetam was finally withdrawn in April 2012 with no seizure recurrence. Due to a clear right-sided hemidystonia, levo-dopa with carbidopa in increasing dose was initiated in May 2012.

A brain MRI was performed at 3 years of age, and revealed subtle signs of cortical atrophy, hypoplastic corpus callosum, T2 and FLAIR high signal abnormalities involving the brain white matter, more prominent in the left hemisphere. Although neurological improvement could be observed, the patient showed a persistent right-sided hemidystonia, increased muscle tone in lower limbs with pyramidal signs and sleep pattern discharges.

## Aim

To present the pharmacological response and therapeutic drug monitoring (TDM) of a paediatric patient with a severe encephalopathy refractory to pharmacological treatment, carrying a genetic variant in KCNT1 gene, whose identification by Next Generation Sequencing (NGS) led to include QND as antiepileptic drug.

## Case Report

To summarize, the initial years of the patient included epileptic encephalopathy development, cognitive and physical impairment which were part of an undiagnosed syndrome resistant to pharmacological treatment.

On 2013, when the patient was 4 years old, a Whole Exome Sequencing (WES) was performed. The entire exome was captured using reagent that targets coding exons from the consensus coding sequence. Capture enrichment was followed by sequencing on Illumina platform using previously described standard protocols. Illumina sequence analysis was performed using the Human Genome Sequencing Center’s integrated Mercury pipeline (Baylor College of Medicine, United States of America). A pathogenic genetic variant associated to EIMFS (Barcia et al., 2012; The ILAE Genetics Commission Blog, 2020 KCNT1 – this is what you need to know, 2020) located in exon 24 (2795 T > C) of the KCNT1 gene (Potassium Sodium-Activated Channel Subfamily T Member 1) was identified.

Due to the remaining nocturnal activity despite many pharmacological schemes, a different approach was implemented.

At that moment new clinical studies showed benefits in using QND in epilepsy (Mikati et al., 2015), due to its activity as KCNT1 blocker. Due to its pharmacological effect and the variant described in the patient, at the age of six years old (in 2015), QND was included as “off label” drug regimen, besides TPM (175 mg/day) and nitrazepam (1.5 mg/day).

Because QND toxicity was well described it was needed to monitor the cardiac function and plasma concentrations. For this aim our laboratory developed and validated HPLC-UV method to quantify QND in human serum. Propranolol was added to the samples as an internal standard, followed by liquid extraction with cyclohexane and evaporation. The chromatography condition included a reversed phase column and a mobile phase consisting of acetonitrile and phosphate buffer (25:75).

Validation parameters studied were specificity (no interferences were found), lineality (0.5–8.0 mcg/ml), repeatability, accuracy (less than 10% CD), sensitivity and stability (in different conditions and time periods). Tests were evaluated according US FDA Guidance for Industry Bioanalytical Method Validation (US FDA Guidance for Industry Bioanalytical Method Validation, 2018).

During QND dose titration, TPM was decreasing the daily doses (initially 175 mg/day) to complete withdrawal, while nitrazepam dose regimen of 2 mg/day was maintained.

TDM was implemented, to establish the individual therapeutic range, as well as electrocardiogram (ECG) studies to assist cardiac follow up. When QND dose was above 50 mg/kg/day in four administrations (achieving a minimum serum level of 1.0 mcg/ml) the therapy started to modify the discharges profile. Only when doses reached 65–70 mg/kg q. i.d (and serum levels were above 1.5 mcg/ml) the patient was seizure free.

According to the original strategy, TPM was almost withdrawn. Surprisingly, QND doses of 65–70 mg/kg which had initially shown therapeutic effectiveness, failed to gain seizures control. Due to this observed synergic effect between TPM and QND, TPM was again indicated. TPM maintenance dose was 2.5 mg/kg/day and then increased to 4.5 mg/kg/day (which achieved levels of 3 mcg/ml and 6 mcg/ml of TPM respectively).

Table 1 shows the variability of QND levels at higher doses compared with lower doses. Initially, when doses increased from 240 to 640 mg/day, QND concentration augmented from 0.6 mcg/ml to 1.0 mcg/ml. But analyzing the same increment in dose (approximately 400 mg/day) with higher doses (from 1,160 to 1,600 mg/day), it resulted in a higher variation of serum levels obtained (1.5–4.1 mcg/ml).

**TABLE 1:** Comparison of different variation in lower and higher doses, occurred during patient follow up with TDM. Levels are more disperse in association with doses increment.

Range of dosesmg/day	Doses variation(Max–min)	Range of plasma concentrationmcg/ml	Plasma concentration variation(Max-min)
240–640	400.0	0.6–1	0.4
1,160–1,600	440.0	1.5–4.1	2.6

It is important to mention that the patient received different pharmacological treatment according particular necessities, such as: antimicrobial agents or laxatives, which might affect QND levels. Moreover, changes in TPM doses could contribute to the variability observed. At the beginning of the treatment the increase in QND doses did not correlate with a proportional increase of QND plasma levels. After a reduction in TPM dose regimen below 50 mg/day the increase in QND doses yielded a proportional dose-concentration relationship.

It is also known that QND cardiotoxicity could produce arrhythmia due to prolongation of QT segment. Our patient experienced the risk of suffering this adverse event on several occasions. Nevertheless, we identified two different related patterns (Table 2). The first one was observed during the titration period, when QND doses were incremented every 14 days. QT segment values corrected themselves without any medical intervention, once normalized titration could continue. On the other hand, when the levels reached were higher than 4.0 mcg/ml, the prolongation was persistent, and it was mandatory to reduce the doses. To sum up, Graphic 1 shows QND levels achieved during the follow-up, correlated with QND doses.

**TABLE 2:** Serum levels and doses related to QT segment prolongation (Reference <0.46 s). In bold is highlighted those situations where reducing doses was necessary to correct QT segment prolongation.

Datedd/mm/yy	Dose(mg/day)	Plasmatic level(mcg/ml)	QT segment(sec.)
22/03/16	560	0.5	0.48
**08/05/17**	**1,320**	**4.0**	**0.60**
06/09/17	1,360	3.0	0.46
22/09/17	1,320	2.8	0.52
16/01/18	1,400	1.9	0.46
25/09/18	1,440	1.8	0.52
21/01/19	1,560	3.4	0.50
**20/08/19**	**1,560**	**5.9**	**0.50**

Values in bold indicate toxicity events.

## Discussion

Although pathogenic variants on *KCNT1* are rare in the population, it has been proposed as an important cause of epilepsy with a wide phenotypic spectrum (Barcia et al., 2012). We characterize the genotype, as a pharmacological target, but also a therapeutic drug monitoring strategy to optimize the pharmacological treatment with the potassium channel blocker QND in association with other antiepileptic drugs in a pediatric patient.

KCNT1 encodes the largest potassium channel subunit and is thought to regulate the hyperpolarization that follows repetitive firing. Functional studies have shown that genetic variants in the cytoplasmic C-terminal domain caused constitutive activation of the potassium channel (Milligan et al., 2016), which modify normal neuronal firing and directly led to epileptogenesis.

A KCNT1 variant previously reported as pathogenic (Vanderver et al., 2014; Yang et al., 2014) was identified in the patient. This ‘*de novo*’ variant is highly conserved, and the p.F932I change is, in principle, the most damaging non-synonymous change associated to this variant.

The KCNT1 variant identified in our work is located immediately adjacent to two previously described pathogenic variants (pArg928Cys and pAla934Thr), and therefore was proposed as the underlying disease-causing mechanism in this case. KCNT1 related epilepsies is included in a broader group of potassium channel related epilepsies that may be amendable to channel specific therapies, such as QND.

QND an old antiarrhythmic and antimalarial drug, has emerged as a potential precision therapy for KCNT1-related epilepsy. *In vitro* functional assays have demonstrated that gain of-function effects associated to KCNT-1 gene variants can be reversed by QND. Although previous case reports showed a significant reduction in seizure burden (Bearden et al., 2014; Milligan et al., 2014), subsequent reports demonstrated less favorable results, raising questions about QND effectiveness for treating KCNT1-related epilepsy (Chong et al., 2016; Numis et al., 2018).

In this manuscript we described the pharmacological effects and therapeutic drug monitoring strategy applied after including QND to a patient’s drug regimen who carries a KCNT1 variant, receiving also TPM and nitrazepam as pharmacological therapy.

One interesting point to note is that in a previous study, a different pattern of pharmacological response was reported related to the KCNT-1 variant identified. In our work, the less frequent 2795 T > C variant resulted in sustained seizure freedom after quinidine treatment, in agreement with the data showed by Fitzgerald et al. (2019) in a patient treated with QND and receiving TPM and nitrazepam as antiepileptic drugs.

It has been also proposed that the limited efficacy of QND previously reported in KCNT1 patients could be related to the limited bioavailability of QND in the CNS. In this way, it has been shown in healthy volunteers that QND levels in CSF could be lower than in plasma where QND CSF concentration were 16% of unbound serum concentrations (range, 4–37%) (Ochs et al., 1980). Anyway, there is no data available of CSF levels in KCNT1 patients receiving QND to evaluate the CSF/plasma quinidine concentration relationship and antiepileptic response.

Moreover, the QND dose increment at higher doses resulted in greater variability in QND plasma levels. This phenomenon could in part be explained by the rise in QND free fraction in the range of concentration observed (1.5–4.0 ug/ml), that could be available to be eliminated or re-distributed according to different factors affecting the patient (Ochs et al., 1980). In this sense, the variability observed, justified the QND monitoring of plasma levels with the aim to optimize dose regimen.

In the largest cohort of patient with KCNT1 related epilepsy, that included 43 patients, QND treatment resulted in a >50% reduction in seizure frequency in only 20% of patients receiving this therapy, with sustained seizure freedom occurring rarely (Fitzgerald et al., 2019). In the study mentioned, therapeutic blood levels were not achieved in many patients, with 45% of patients failing to reach a blood level of 2 ug/ml.

There are several potential explanations for this finding, but we can hypothesize that QTc prolongation concern, infrequent use of this drug or lack of knowledge regarding the most appropriate strategies for therapeutic monitoring, could be some of the reasons that prevented reaching higher therapeutic levels.

TDM is a useful tool when allows us to select a goal and to reach a target by developing a pharmacokinetic/pharmacodynamics (PK/PD) model for a particular patient, but it is useless when there is a lack of skills related to drug monitoring practice, because blood monitoring of a particular drug is very infrequent, or if no pharmacokinetics recommendations are given.

In our study QND drug regimen optimization was performed and a high proportion of QND levels reached the therapeutic target. During the follow-up, intensive therapeutic drug monitoring was accompanied by pharmacokinetics recommendation of QND dose adjustment with a high rate of physician compliance. This strategy allowed to modify QND dose regimen to achieve plasma levels into the individual therapeutic range for this patient.

To highline from our work, the patient got free from seizures receiving QND 70–90 mg/kg q. i.d, TPM 4.5 mg/kg/day and nitrazepam 2.5 mg/day as pharmacological treatment. Due to the fact that QND effectiveness decreased when TPM doses were below 20–30 mg/day during TPM withdrawal, and that QND and TPM does not share the same mechanism of action, we can suggest a potentially synergic effect in this case. Initially, high doses of TPM showed to be ineffective to control seizures in this patient. However low doses of TPM were needed associated to QND to get free from seizures. Although the nature of this interaction needs further studies, QND as monotherapy may not be as effective acting at the KCNT1 receptor level making necessary the association of another antiepileptic drug to maintain the pharmacological response.

From a pharmacokinetic point of view QND is a very potent inhibitor of the efflux transporter P-glycoprotein (PGP), and is involved in the increased bioavailability at the CNS and toxicity of different drugs. It has been shown that TPM is a substrate of PGP, suggesting that up-regulation of MDR-1 gene could affect the TPM brain levels. QND could be acting as a “booster” and might be increasing TPM levels that reach CNS (Sadeque et al., 2000), allowing the use of lower doses of TPM that showed to be effective.

In our experience QND have shown to be affected by other conditions. The patient levels drop off on many occasions neither for changes in doses nor changes in the administration of QND. Levels that were below expected could be associated for example to other pharmacological treatment such as wide spectrum antibiotics which might have affected QND absorption. Laxatives also may have decreased QND absorption because of the increment in bowel motility.

Increasing TPM doses are also suspected of altering serum QND concentrations through several different mechanisms that need to be studied more carefully. According to bibliography, the inhibition of carbonic anhydrase enzyme by TPM (Ruiz Granados et al., 2015) might conduct to metabolic acidosis, producing an increase in urinary pH and QND plasma levels. By the contrary, in our work at higher TPM doses, QND titration was not accompanied by a proportional increase of QND plasma levels until TPM regimen was below 50 mg/day. Anyway, as we mentioned, the fluctuation of QND levels was more significant at higher QND doses, and TDM was successfully applied in order to reach the individual therapeutic range that was defined between 1.5–4.0 ug/ml for this patient.

Considering previous studies and the results presented in this report, we suggest that in patients with KCNT-1 related epilepsy, the inclusion of QND should be accompanied of the analysis of the KCNT-1 genetic variants identified, together with a TDM strategy that allows to characterize the pharmacokinetic of QND, with the aim to reach the individual therapeutic target in order to increase efficacy with minimal side effects.

## Conclusion

The implementation of Precision Medicine by using tools such as NGS and TDM led to diagnose and select a targeted therapy for the treatment of a KCNT1-related epilepsy in a patient presented with EIMFS in early infancy and poor response to antiepileptic drugs. QND an old antiarrhythmic drug, due to its activity as KCNT1 channel blocker, associated to TPM resulted in seizures control. Due to high variability observed in QND levels, TDM and pharmacokinetic characterization allowed to optimize drug regimen to maintain QND concentration between the individual therapeutic range and diminish toxicity.

## Data Availability

The raw data supporting the conclusions of this article will be made available by the authors, without undue reservation.
